# Optimization of Alkali Treatment for Production of Fermentable Sugars and Phenolic Compounds from Potato Peel Waste Using Topographical Characterization and FTIR Spectroscopy

**DOI:** 10.3390/molecules28217250

**Published:** 2023-10-24

**Authors:** Qudsia Mushtaq, Nicolas Joly, Patrick Martin, Javed Iqbal Qazi

**Affiliations:** 1 Microbial Biotechnology Laboratory, Institute of Zoology, University of the Punjab, New Campus, Lahore 54590, Pakistan; mushtaqqudsia@yahoo.com; 2 University Artois, Unilasalle, ULR7519—Unité Transformations & Agro-Ressources, F-62408 Béthune, France; nicolas.joly@univ-artois.fr

**Keywords:** sodium hydroxide (NaOH), potato peel waste (PPW), phenolic compounds, scanning electron microscopy, fourier-transform infrared spectroscopy, Box-Behnken design

## Abstract

Potato peel waste (PPW) was utilized as a bio-template for the production of valuable compounds such as reducing sugars (RS), total sugar (TS) and total phenolic compounds (TPC). Two methods of alkali treatments, i.e., chemical (NaOH) and thermochemical (NaOH assisted with autoclaving) processes, were employed for the deconstruction of PPW. Response surface methodology (RSM) was used to study the effects of alkali concentration (0.6–1.0 *w*/*v*), substrate concentration (5–15 g) and time (4–8 h) on the extraction of RS, TS and TP from PPW. The application of alkali plus steam treatment in Box-Behnken design (BBD) with three levels yielded the optimum releases of RS, TS and TP as 7.163, 28.971 and 4.064 mg/mL, respectively, corresponding to 10% substrate loading, in 0.6% NaOH for 8 h. However, the alkali treatment reported optimum extractions of RS, TS and TP as 4.061, 17.432 and 2.993 mg/mL, respectively. The thermochemical pretreatment was proven a beneficial process as it led to higher productions of TP. FTIR and SEM were used to analyze the deterioration levels of the substrate. The present work was used to explore the sustainable management of PPW, which is a highly neglected substrate bioresource but is excessively dumped in open environment, raising environmental concerns. The cost-effective methods for the breakdown of PPW starch into fermentable sugars might be utilized to extract valuable compounds.

## 1. Introduction

Due to severe environmental concerns related to the dumping of food wastes, such as microbial spoilage and the emission of harmful gases, such practices are being strongly discouraged. The exploitation of food wastes for alternative and sustainable applications are being investigated. PPW can be transformed into various valuable products such as biofuel, biogas, bio-fertilizers, bio-absorbents, reducing sugar, enzymes and antioxidants [1,2,3]. About one-third of the total food produced for human consumption is estimated to be wasted [4], and these losses were estimated to cost 1 trillion USD [5]. Potato (*Solanum tuberosum*) is a major vegetable crop with annual production of 370 million tons [6]. A major part of potato production is as a fresh product, whereas the remaining part is used to meet the demands of fast food consumption such as frozen fries, crisps, starch and dried potatoes. During potato processing, 15 to 40% of the initial weight is descaled and is termed as PPW [7,8].

PPW is the major neglected by-product in the potato-processing industry, whilst it represents a potential source of valuable compounds. Among them, polyphenols are important precursors for natural antioxidants and offer tremendous application potential owing to their antioxidant and antimicrobial properties. Approximately 50% of these molecules are present in the peel and adjacent sections [9,10]. Synthetic antioxidants are extensively used in food and pharmaceutical applications and have been reported to induce various chronic diseases. Natural antioxidants are highly appealing to various industrial sectors and offer protective applications such as the prevention of oxidative damage and cardiovascular diseases [11]. PPW has an important nutritious profile, containing starch, dietary fibre and protein. The most abundant nutrients in PPW are carbohydrates, accounting for up to 88 g/100 g dry weight. The amount of starch accounts for up to 52% of total carbohydrate content. PPW also has a considerable amount of dietary fibre, which can regulate intestinal health by lowering the cholesterol effect [8,12,13,14,15]. Vázquez et al. [16] have recently documented the promising potential of utilizing waste materials, specifically Garnacha Tintorera bagasse and potato residues, as sustainable substrates for the co-production of valuable bioproducts, such as bacterial cellulose and gluconic acid.

Unprecedented rapid urbanization and continuously increasing standards of living in modern society lead to the production of huge quantities of solid food wastes. Consequently, additional management practices for the management of this waste exerts strong pressure on the economy and society [17,18]. There are different waste management practices for the disposal of food waste, but they have many problems such as the production of toxic compounds, high costs and environmental pollution [19]. The familiar waste disposal methods are landfills, incineration, composting and thermochemical processes [19,20]. The most common method of waste disposal is landfill, which produces greenhouse gases and toxic heavy metal compounds that contaminate land and ground water. This approach also requires large land area and a long time for disposal [21,22,23,24].

Incineration is a relatively rapid method for waste management and requires small land area but leads to the production of combustion residuals including bottom and fly ash. The elements contained in ash cannot be degraded and are eventually enriched in living organisms [25,26]. Composting is an effective method of degrading wastes into products that can be used safely as biofertilizers and soil improvements [27,28,29]. However, various challenges are also associated with this practice, including the release of CO_2_, the depletion of O_2_, climate change and offensive smells [30]. Biotechnological upgradation appears promising for the concomitant provision of low-cost substrates and safe solid food waste management. The application of PPW for microbial mediated saccharification and the provision of other useful products is representative of such efforts.

Fermentable sugars can be obtained from PPW which can boost biofuel production. PPW is a rich source of primary metabolites, which principally include carbohydrates, which are prone to undergo fermentation to produce ethanol, lactic and acetic acid [31]. The breakdown of complex starch polymers into small monomeric sugars makes it more accessible to improve the production of biofuel. PPW conversion into biofuel and biogas offers excellent alternatives to fossil fuels. There are many previous research reports which have confirmed the conversion of PPW to biofuels [32,33,34,35].

A few new methods such as ultrasonic and microwave assisted extractions [36,37], pressurized liquid-based removal [38] and acidified solvents-based extraction [39] have been documented to isolate the valuable compounds from PPW. These are, however, highly complex and expensive methods; therefore, the need for simple, low cost and time saving methods cannot be over-emphasized. Alkali hydrolysis offers a number of advantages such as a simple pretreatment for substrate, easily available and cost-effective NaOH and low reaction temperature. Little information is available about the kinetics and efficiency of the alkali hydrolysis of PPW into sugars. In the present work, the efficacy of alkali and alkali-assisted autoclaving was clearly observed.

Alkali and alkali-assisted steam treatment to extract useful compounds from different plant biowastes are methods of choice in select situations to obtain value added products [40,41,42,43]. The treated substrates are compared with their respective controls by SEM to assess the level of polymeric destruction [44,45,46].

The present study aimed to optimize the alkali treatment of PPW for yielding RS, TS and TPC using a Box–Behnken design with three levels. The model developed in this study is cost-effective for the bioconversion of PPW into value added products such as fermentable sugars and phenolic compounds. As regards the optimum concentrations of fermentable sugars for bioethanol fermentation, they can easily be adjusted either by the evaporation of the treated fluid or by its dilution to correspond to the requirements of ethanologenic microbe(s). Evaporation is economically affordable in country such as Pakistan, where solar insolation is available throughout the year. However, other means to improve the saccharification efficiencies of the procedures reported here, such as varying steam pressures, might be employed to increase the yields. Soni et al. [47] have explored various conditions by using different alkali and acid concentrations to treat PPW and assessing their potential to produce bioethanol. They reported 368.53 mg/g of RS after 72 h using alkali-treated PPW, and after enzymatic hydrolysis ethanol concentration was recorded as 19.9g/L. Another research reported 88.2 g/L RS after enzymatic hydrolysis with 20% PPW. The maximum ethanol concentration of 23.3 g/L was produced under optimum conditions [48]. This effort is promising for the provision of cheaper substrates for various biotechnological processes such as the production of second-generation bioethanol. Accessory benefits include urban food waste management and the utilization of single-cell enriched fermentation residues as animal feed supplement.

## 2. Results and Discussion

The pretreatment of the substrates rich in carbohydrates is performed to breakdown the highly branched polymers and yield the maximum sugars. The present work was designed, as described below, to release maximum RS, TS and TP.

### 2.1. RSM Optimization and Validation

In this study RSM was used to optimize the pretreatment conditions using PPW as cheap biowaste. BBD was conducted using substrate concentration, NaOH concentration and time as independent variables. The alkali and alkali-plus-steam treatment of PPW was carried out with three variable factors as presented in Table 1. The experimental runs were performed according to the BBD, and all experiments were run in triplicates. The observed and predicted values of RS, TS and TP after alkali and alkali-assisted autoclave treatment are shown in Table 2 and Table 3. Multiple regression analysis was applied, and second-order polynomial regression equations (Equations (1)–(6) revealed the influence of three variable factors (X_1_, X_2_ and X_3_) on the extraction yield of useful compounds from PPW.

In the case of alkali hydrolysis, the maximum extraction of RS, TS and TP was measured as 4.061, 17.432 and 2.993 mg/mL, respectively, corresponding to 10 g of PPW, 0.8% of alkali concentration and 8 h (Table 2). In the case of alkali-plus-steam hydrolysis, maximum extraction of RS, TS and TPs was measured as 7.163, 28.971 and 4.064 mg/mL, respectively. The experimental conditions for optimum yield were observed at run No. 9, 7 and 14 (Table 3). The thermochemical treatment proved more effective in the monomerization of polymeric sugars of PPW. Several studies have indicated higher yields of useful compounds from the alkali and alkali-plus-steam treatment of PPW [47,49,50,51,52].

#### 2.1.1. Significance by ANOVA

The linear and the quadratic effects of the variables studied and their interaction on the response yield was observed using analyses of variance (ANOVA). The significance was revealed by Fisher’s F-test and the probability value. In the case of alkali hydrolysis, F-test values of 31.64, 16.33 and 10.79 and *p*-values of 0.002, 0.010 and 0.022 were observed for RS, TS and TP, respectively. The results revealed that the concentration of the substrate and time were the most significant parameters during the pretreatment process. For RS production, X_1_, X_3_, X_1_^2^ and X_3_^2^ were found to be significant as their *p*-values were calculated as 0.006, 0.014,0.013 and 0.002, respectively, while for TS production, X_1_^2^ and X_3_^2^ were significant as their *p*-values were calculated as 0.056 and 0.010, respectively. In the case of TPC, the factors X_2_, X_3_^2^ and X_1_ × X_3_ were found to be the most significant variables as their *p*-values were noted as 0.032, 0.031 and 0.022, respectively (Table 4).

The regression equations for chemical treatment are as follows:

Y (RS mg/mL) = 5.4990 − 0.4874X_1_ − 6.526X_2_ + 1.1359X_3_ + 0.0125X_1_^2^ + 4.6760X_2_^2^ − 0.1172X_3_^2^ + 0.0765X_1_X_2_ + 0.0132X_1_X_3_ − 0.0593X_2_X_3_ (1)

Y (TS mg/mL) = 0.678 − 3.485X_1_ + 119.314X_2_ − 9.209X_3_ + 0.122X_1_^2^ − 59.602X_2_^2^ − 1.244X_3_^2^ + 0.759X_1_X_2_ − 0.011X_1_X_3_ − 5.421X_2_X_3_ (2)

Y (TP mg/mL) = −2.9351 + 0.0734X_1_ + 12.326X_2_ − 0.3487X_3_ + 0.0090X_1_^2^ − 4.8771X_2_^2^ − 0.0770X_3_^2^ − 0.0968X_1_X_2_ − 0.0326X_1_X_3_ − 0.4325X_2_X_3_ (3)

In the case of alkali-plus-steam hydrolysis, F-test values of 17.99, 42.1461 and 24.08 and probability values of 0.008, 0.030 and 0.004 were observed for RS, TS and TP, respectively. The interactive effect of X_2_ × X_3_ and X_1_ × X_3_ on the response yield was found to be very significant. The results revealed that the concentration of PPW and time (h) were the most significant parameters during the pretreatment process. For RS yield, X_2_ and X_2_ × X_3_ were found to be significant factors as their *p*-values were recorded as 0.034 and 0.008, respectively, while X_1_ × X_3_ (0.030) was significant for the production of TS. In the case of TPC, the factors X_3_^2^ and X_2_ × X_3_ were found to be the most significant variables as their *p*-values were measured as 0.010 and 0.004, respectively (Table 5).

The regression equations for thermochemical treatment are as follows:

Y (RS mg/mL) = 28.8530 − 0.1261X_1_ − 43.260X_2_ − 2.019X_3_ − 0.0039X_1_^2^ − 13.4156X_2_^2^ − 0.0972X_3_^2^ + 0.2490X_1_X_2_ + 0.0197X_1_X_3_ + 3.4856X_2_X_3_ (4)

Y (TS mg/mL) = 13.540 + 0.1115X_1_ − 44.222X_2_ + 7.4944X_3_ + 0.0598X_1_^2^+ 26.4677X_2_^2^ − 0.0299X_3_^2^ + 0.3935X_1_X_2_− 0.3246X_1_X_3_ − 2.8856X_2_X_3_ (5)

Y (TP mg/mL) = 0.8233 + 0.0777X_1_ − 1.3983X_2_ + 0.6086X_3_ − 0.00384X_1_^2^ − 07.3541X_2_^2^ − 0.1671X_3_^2^ + 0.1747X_1_X_2_ − 0.00150X_1_X_3_ + 1.9293X_2_X_3_ (6)

The model’s goodness of fit was determined by the coefficient of determination (R^2^ value).The R^2^ values in the case of alkali hydrolysis were measured as 97.52%, 90.76% and 93.61% for RS, TS and TP, respectively, while in the case of alkali-assisted autoclave treatment these values were measured as 87.14%, 93.25% and 94.45% for RS, TS and TP, respectively. The adjusted R^2^ values were measured as 74.12%, 93.05% and 82.11% for chemical treatment, while for thermochemical treatment the same values were measured as 64.00%, 81.11% and 84.46% for RS, TS and TPC, respectively, explaining the credibility of the model. Furthermore, the predicted R^2^ values were measured as 0.71, 0.79 and 0.80 for chemical treatment, while for thermochemical treatment same values were measured as 0.68, 0.76 and 0.75 for RS, TS and TPC, respectively, explaining the credibility of the model.

#### 2.1.2. Contour Plots

**Figure 1:**
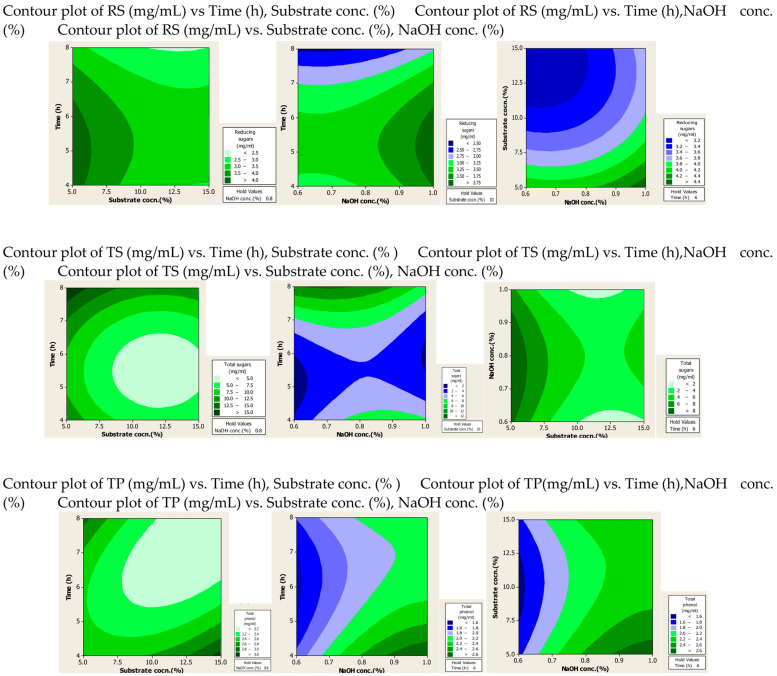
Contour plots after alkali treatment of PPW.

**Figure 2:**
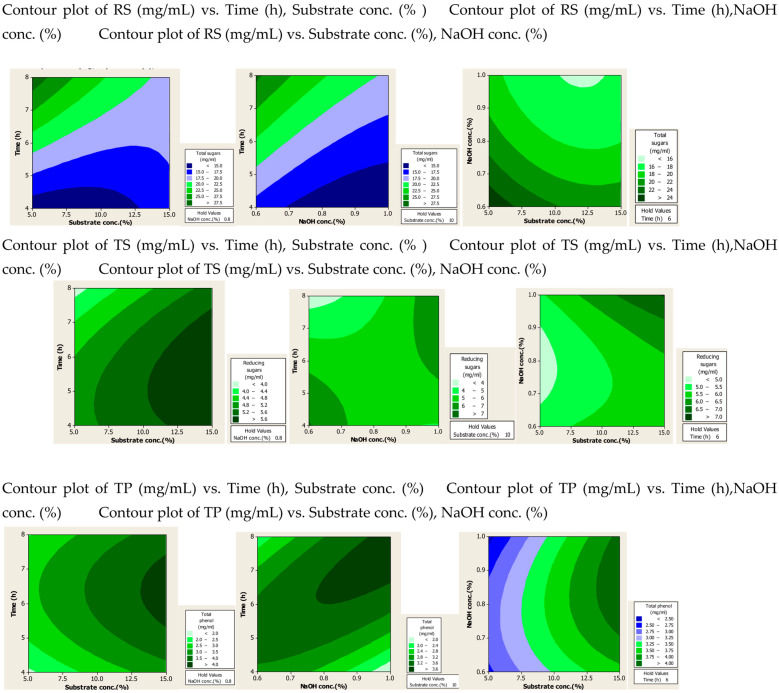
Contour plots after alkali assisted autoclave treatment of PPW.

The effect of the interaction of different factors on the extraction of RS, TS and TPC after alkali and alkali-assisted autoclave treatment was illustrated by 2D contour plots, as shown in Figure 1 and Figure 2. In these plots three main colours are shown: light green and blue represent the lowest yield, while dark green represents the highest yield. From the alkali-treated contour plots it can be observed that substrate and alkali concentration had a direct relation to the production of RS, whereas time had an inverse relation to the production of RS. For TS production, optimum values were observed at high values of substrate and alkali concentration and time. High concentrations of substrate and alkali were favourable for the production of phenolic compounds, whereas time did not significantly affect the yield of phenolic compounds.

From the alkali-plus-steam-treated contour plots it is evident that high RS production occurred at lower values of three parameters. For TS and TP production none of the three parameters was found significant, but increased production was observed with high values of substrate and alkali concentration.

### 2.2. FTIR Analysis

**Figure 3:**
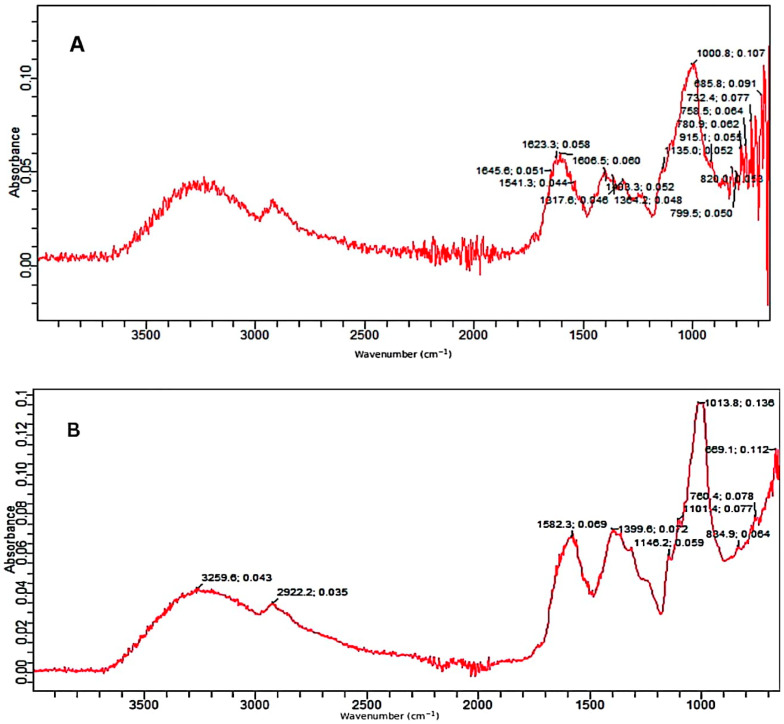

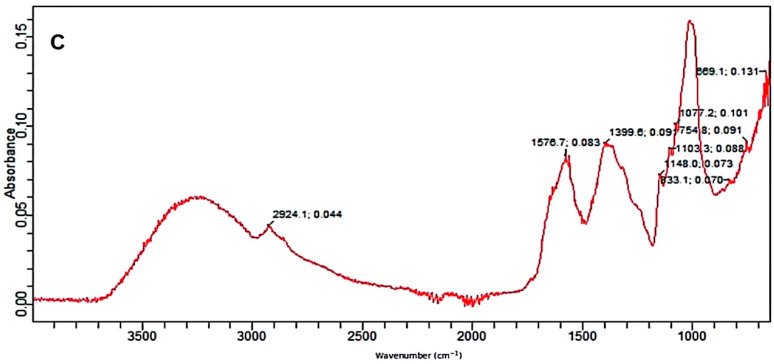
FTIR absorption spectra; (**A**) control, (**B**) alkali-treated and (**C**) alkali-plus-steam-treated PPW.

The control as well as the pretreated substrates were analyzed by the FTIR facility. The FTIR (Figure 3A–C) confirmed the deterioration of various bonds in treated samples of PPW. FTIR spectra of alkali and alkali-assisted autoclave-treated PPW ranged from 4000 to 500 cm^−1^. Both spectra revealed a band between 3000 and 3300 cm^−1^, which confirmed the presence of a free and bounded -OH stretching vibration derived from degraded polymers such starch, cellulose and lignin. In the control sample, there were more peaks, reflecting the complexity of different polymers and bonds which apparently deteriorated in the treated samples. The intensity of different peaks was also high in the control sample (Figure 3A).

In the alkali-treated PPW, the spectrum displayed the characteristic peak at 2922 cm^−1^ which can be attributed to the carbon hydrogen (C–H) bond stretching of methyl and methylene groups in the polymers present in PPW. The absorption peak at 1582/1541 is attributed to C–H bond deformation in polymers such as starch and lignin present in PPW which could be highly increased in alkali-treated PPW as compared to untreated substrate. The peaks at 1399/1317 cm^−1^ revealed C–H deformation in various polymers such as starch, lignin and cellulose which was significantly increased in treated PPW as compared to untreated PPW. The bands at 1146 and 1101 cm^−1^ reflected carbon oxygen bond (C–O–C) stretching in different polymers (starch and non-starch polysaccharides) existing in PPW (Figure 3B).The bands at 1013, 834 and 760 cm^−1^ depicted the deformation of various compounds such as polysaccharides and aromatic and halogen compounds [53].

The FTIR spectrum of alkali-assisted autoclaved PPW showed a very broad band between 2700 and 3300 cm^−1^, which can be assigned to aldehydes or acids formation (benzoic or carboxylic acid). The absorption peak at 1576/1541 cm^−1^ is attributed to the C–H bond deformation of polymers existing in PPW, which increased in alkali-plus-autoclave treatment as compared to untreated substrate. The peak at 1399/1346 cm^−1^ depicted the C–O deformation and confirmed more degradation in alkali-assisted autoclaving treatment. The bands at 1148 and 1103 cm^−1^ described the C–O–C vibrations in various components of PPW (Figure 3C). The band at 1077 denoted the C–O vibrations in various alcoholic groups of cellulose. The bands at 833, 754 and 699 cm^−1^ reflected the C–H deformation of lignin component [54,55]. Similar recordings of band patterns have been reported by several research reports [56,57].

### 2.3. Morphological Changes in Untreated and Alkali plus Steam Treated PPW

**Figure 4:**
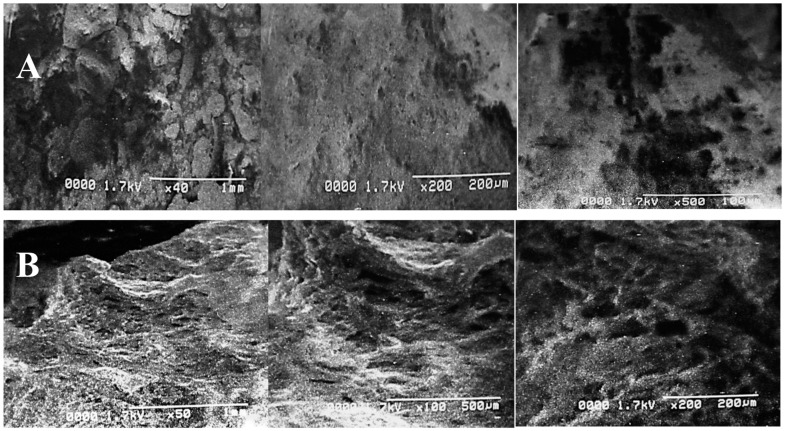
Scanning electron micrographs of PPW of control (**A**) and alkali-plus-steam-treated PPW (**B**).

The structural variations and deformations in PPW after both treatments were clearly revealed by SEM, as shown in Figure 4. The alkali reaction destroyed the structural bonding by creating some irregular pores and cracks. Electron micrographs depicted a significant difference between the surface structures of the control and treated samples. The untreated PPW structure displayed a complex and intact order without any evident fragmentation (Figure 4A). However, alkali-assisted steam treatment deteriorated the compactness of the structure, resulting in cracks, pores and damage to biomass (Figure 4B). Similar findings and observations have been reported in the study by [56]. They have confirmed the production of deformations, porosity, cracks and crevices in the SEM images of nanoparticle pre-treated (NAAP) PPW. Ghazanfar et al. [58] have reported a disrupted and perforated biomass structure after NaOH treatment using SEM images. Goyi et al. [59] have also reported a perforated structure of PPW after using the hydrothermal carbonization (HTC) extraction technique while evaluating its potential for water treatment. Similar findings have also been reported in research reports while evaluating the potential of PPW for different applications [60,61].

### 2.4. Degradation Index of Raw and Treated PPW

**Figure 5:**
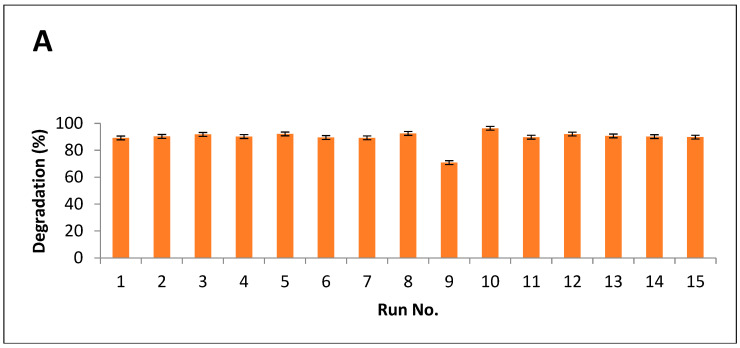

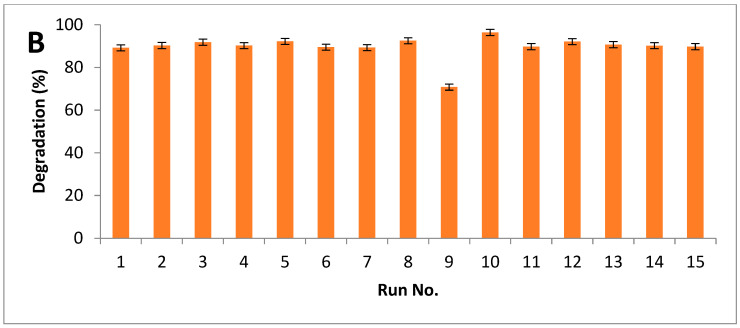
Degradation index of PPW (**A**) alkali treatment (**B**) alkali plus steam treatment.

Results indicated that maximum breakdown after alkali hydrolysis was measured as 94%, while 96% of degradation was observed after alkali-plus-steam treatment (Figure 5A,B).

### 2.5. Alkali Saccharification of PPW

**Figure 6:**
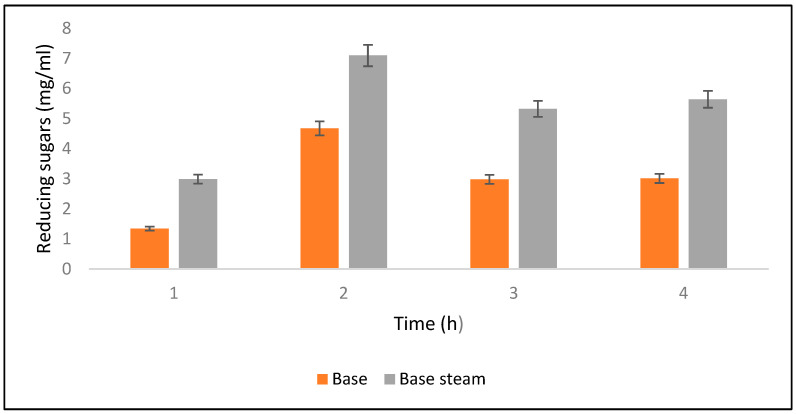
Yields of reducing sugars released after alkali and alkali-plus-steam hydrolysis from the PPW post treatments.

Saccharification was checked at different time intervals such as 4, 6, 8, and 10 h after alkali hydrolysis. Results revealed that maximum saccharification (0.70%) was obtained after 6 h of alkali-assisted autoclave treatment, whereas minimum saccharification (0.029%) was observed after 10 h. Meanwhile, for alkali-treated PPW maximum saccharification was observed as 0.013% (Figure 6). Ben Taher et al. [62] have reported a high saccharification yield using alkali treated PPW.

## 3. Materials and Methods

### 3.1. Substrate

In Pakistan, as in many other countries, potatoes represent one of the major staple foods. Their peels, however, are usually discarded [63,64,65]. PPW were obtained from a local fries shop (Gate No.4, Quaid-e-azam campus University of Punjab, Lahore, Pakistan). They were properly washed to remove all dirt, sun dried for 24 h and oven dried at 50 °C for 48 h until consistent weight. They were powdered by an electric mill to obtain a uniform size of 1 mm. The PPW was stored in air tight containers at room temperature (25 ± 5 °C) until use.

### 3.2. Pretreatment

**Figure 7:**
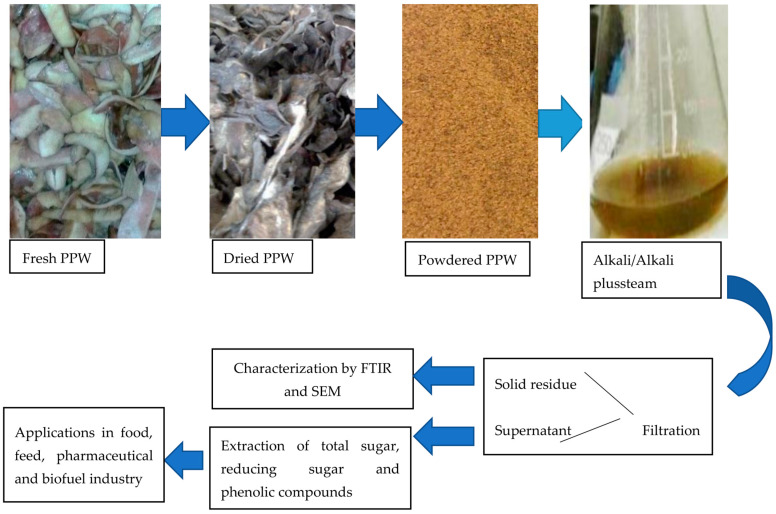
A schematic overview of value added compounds from PPW.

Pretreatment was carried out using two methods, chemical (NaOH) and thermochemical (NaOH-assisted autoclaving).All experiments were carried out in 250 mL Erlenmeyer flasks with a working volume of 100 mL, in triplicates. Different concentrations of substrate were soaked in dissimilar concentrations of NaOH according to the experimental design for different time periods. Following the soaking process, the pretreatment reactors were subjected to pressurized heat at 121 °C, 21 psi for 15 min. Following the treatment process, all samples were filtered, and clear liquid was used to assess the value of RS, TS and TPC. The pH value of the clear filtrate was adjusted to approximately 6.0 (Figure 7).

### 3.3. Experimental Design

RSM with three factors and three levels were used to optimize pretreatment process in this work. The independent factors used were substrate concentration (X_1_), NaOH concentration (X_2_) and time (X_3_), which were varied in the range of 5–15 g, 0.6–1.0% and 4–8 h, respectively (Table 1). A total of 17 experiments were carried out in triplicates. The experimental data were fitted in the quadratic polynomial model equation relating the input parameters to yield.

The yield was calculated from the following equation using the Minitab software (16th version).

*Y = b*_0_*+ b*_1_X_1_*+ b*_2_X_2_*+ b*_3_X_3_*+ b*_11_X_1_^2^*+ b*_22_X_2_^2^*+ b*_33_X_3_^2^*+ b*_12_X_1_ X_2_*+ b*_13_X_1_ X_3_*+ b*_23_X_2_ X_3_ (7)

where *Y* is the response, *b*_0_ is the intercept, *b*_1_X_1_ to *b*_3_X_3_ are linear coefficients, *b_11_*X_1_^2^ to *b_33_*X_3_^2^ are quadratic coefficients and *b_12_*X_1_ X_2_ to *b_23_*X_2_ X_3_ are interactive coefficients.

### 3.4. Analytical Methods

After pretreatment, the filtrate was analyzed for the estimation of RS, TS and TP. Total sugars and reducing sugars released during pretreatment were estimated by [66] and the DNS method [67]. Total phenolic compounds released in the filtrate were estimated by [68].

#### 3.4.1. Determinations of TS and RS

TS and RS released following pretreatment were estimated. Glucose was used as the standard, and a series of glucose solutions (0.05–0.4 mg/mL) was prepared to establish the standard curve. For the analysis of total sugar, 0.5 mL of 5% phenol was added to the 0.5 mL of the sample in each test tube. Afterwards, 2.5 mL of sulphuric acid was added to each test tube, and the contents were shaken well. After 10 min, the test tubes were placed in a water bath at 25–30 °C for 20 min. Thereafter, the colour was read at 490 nm.

For the analysis of RS, 0.5 mL of the sample was taken in a test tube to which 1.5 mL of 3,5-Dinitrosalicylic acid (DNS) was added; it was shaken well and covered with aluminium foil. After 10 min, all test tubes were kept in a boiling water bath for 10–15 min. The colour developed was read at 546 nm.

#### 3.4.2. Determination of TPC

For the estimation of phenolic compounds, a series of gallic acid solutions (0.01–0.35 mg/mL) was used to prepare the standard curve. For the analysis, 0.5 mL of a sample was taken in a test tube to which 2.5 mL of diluted Folin-Ciocalteu (FC) reagent was added. After 5–10 min, 1 mL of 1 M Na_2_CO_3_ solution was added. Test tubes were shaken well and placed at room temperature; the absorbance was then measured at 760 nm.

### 3.5. Infrared Spectroscopy Analysis

The absorption spectra of the treated and untreated substrate were obtained using FTIR spectroscopy (Agilent technologies Cary 630, Santa Clara, CA, USA). The FTIR spectra of the samples were recorded in the range of 4000–400 cm^−1^.

### 3.6. Scanning Electron Microscopy (SEM)

SEM analysis was conducted to examine the size, shape and morphology of the samples and to compare the topographical modifications in the alkali-treated samples with those in the control substrate. SEM analysis was performed using a Jeol JSM-6490LA microscope, Tokyo, Japan. The sample analysis was conducted with an accelerating voltage of 10 kV at a working distance of 15 mm, and the microscope’s beam intensity was set at 11. Sample preparation for SEM involved suspending 1 mg of the powdered potato peel sample in 1 mL of distilled water in a 1.5 mL Eppendorf tube. The suspension was then sonicated for 1–2 min at 60 amplitudes with 0.5 cycles using a probe sonicator. The Eppendorf tube was placed in a −20 °C freezer overnight. The sample suspension was thawed and sonicated again. One hundred μL of the suspension was taken and resuspended in 900μLof distilled water and subjected to another round of sonication. Subsequently, this suspension was placed in a freezer for 3 h. Meanwhile, glass slides were cut into 1 cm^2^ pieces which were then washed with 70% ethanol and placed in an oven at 70 °C for 1 h. The frozen sample was taken out, thawed and sonicated. A drop of the sample was then placed on a heated slide, which was then dried in an oven overnight.

Following this, the sample was sputter-coated with carbon using a sputter coater model (Jeol JFC-1500, Tokyo, Japan). The coated sample slide was affixed to conductive tape stubs and subjected to SEM analysis. SEM images of the potato peel samples were acquired and analyzed at various magnifications, resolutions and angles.

### 3.7. Alkali Saccharification of PPW

Dilute alkali solutions (0.6–1.0 *w*/*v*) were used for PPW hydrolysis. Saccharification was performed using two methods via alkali and alkali-plus-steam treatment. Five grams of PPW and a solution of sodium hydroxide (*w*/*v*) were added in an Erlenmeyer flask with a capacity of 250 mL and covered with aluminium foil. The PPW mixture with specific parameters was kept in a shaking incubator at 100 rpm at 37 °C. The filtration of the hydrolysed mixture was completed using Whatman filter paper No.1. The filtrates were checked after 4, 6, 8 and 10 h. Samples were taken at the same intervals to determine RS. The saccharification (%) was measured by the following formula [69]:

$$Saccharification\;(\%)=\frac{Reducingsugars\;(mg/mL)}{Substrateused\;(mg/mL)}$$

### 3.8. Mass Balance

To calculate mass balance, pretreated liquid was carefully filtered through Whatman filter paper No.1. Afterwards, filter papers containing the substrate were placed in an oven at 50 °C until consistent mass. The following formula was used to calculate solubility index (S.I):

(8) $$Solubility\;index=\frac{Initial\;weight-Final\;weight}{\begin{array}{c} \;Final\;weight\; \end{array}}\;\times \;100$$

## 4. Conclusions

In this work, alkali and alkali-plus-steam treatments were used to extract bioactive compounds from PPW. The alkali-assisted steam treatment was found to be very effective and yielded 4.064 mg/mL of phenolic compounds from PPW. Optimum extractions of RS and TS were measured as 7.163 and 28.971 mg/mL, respectively. The maximum yield of phenolic compounds revealed that the lignin part was degraded. Lignin degradation makes the treated substrate more accessible for enzymatic hydrolysis, which subsequently boosts the saccharification process. The simple and cost-effective strategy of the deconstruction of PPW into valuable products is promising for the provision of various feedstocks to biotechnological processes with the sustainable management of food wastes. The alkaline treatments may render the PPW a cost-effective substrate for various biotechnological processes, whilst the waste microbial content will also be reduced for a better urban environment.

## Figures and Tables

**Table 1:** Codes and levels of the parameters used for BBD.

		Level		
**Factor**	**Code**	−1	0	1
**Substrate conc. (%)**	**X_1_**	5	10	15
**NaOH conc.(%)**	**X_2_**	0.6	0.8	1.0
**Time (h)**	**X_3_**	4	6	8

**Table 2:** Results of BBD on yields of RS, TS and TP (mg/mL) after alkali treatment of PPW.

				Reducing Sugars			Total Sugars			Total Phenol		
Run No.	X_1_	X_2_	X_3_	Observed	Predicted	Residual	Observed	Predicted	Residual	Observed	Predicted	Residual
1	5	0.6	6	4.061	4.151	−0.09	6.481	8.391	−1.91	1.535	1.751	−0.216
2	5	0.6	6	2.911	3.041	−0.13	2.181	1.817	0.364	1.723	1.756	−0.024
3	15	1.0	6	4.675	4.544	0.13	6.116	6.480	−0.364	2.762	2.728	0.034
4	15	1.0	6	3.831	3.740	0.091	4.852	2.941	1.911	2.563	2.346	0.217
5	10	0.8	4	4.196	4.196	0.00	14.432	12.143	2.289	2.676	2.611	0.065
6	10	0.8	4	3.014	2.974	0.04	7.320	7.305	0.015	2.957	3.075	−0.118
7	10	0.8	8	3.148	3.187	−0.039	17.432	17.446	−0.014	2.993	2.874	0.119
8	10	0.8	8	2.496	2.495	0.000	9.883	12.171	−2.288	1.969	2.033	−0.064
9	5	0.6	4	3.253	3.162	0.091	1.944	2.322	−0.378	2.008	1.856	0.152
10	15	1.0	4	3.625	3.755	−0.13	4.341	6.265	−1.924	2.889	2.987	−0.098
11	5	0.6	8	2.596	2.465	0.131	13.667	11.742	1.925	1.912	1.814	0.098
12	15	1.0	8	2.873	2.964	−0.091	7.391	7.012	0.379	2.101	2.252	−0.151
13	10	0.8	6	3.483	3.368	0.115	4.421	4.244	0.177	2.112	2.114	−0.002
14	10	0.8	6	3.264	3.368	0.256	5.206	4.244	0.962	2.101	2.114	−0.013
15	10	0.8	6	3.359	3.368	−0.009	3.107	4.244	−1.137	2.131	2.114	0.017

**Table 3:** Results of BBD on yields of RS, TS and TP (mg/mL) after alkali-plus-steam-treated PPW.

				Reducing Sugars			Total Sugars			Total Phenol		
Run No.	X_1_	X_2_	X_3_	Observed	Predicted	Residual	Observed	Predicted	Residual	Observed	Predicted	Residual
1	5	0.6	6	5.212	5.267	−0.055	25.612	25.685	−0.073	2.710	2.691	0.019
2	5	0.6	6	5.851	5.900	−0.049	22.072	21.645	0.427	3.626	3.659	−0.033
3	15	1.0	6	5.462	5.412	0.05	18.371	18.797	−0.426	2.439	2.405	0.034
4	15	1.0	6	7.097	7.041	0.056	16.405	16.331	0.074	4.054	4.072	−0.018
5	10	0.8	4	4.993	5.012	−0.019	14.134	13.458	0.676	1.963	1.895	0.06
6	10	0.8	4	5.723	5.748	−0.025	16.873	16.697	0.176	3.364	3.243	0.21
7	10	0.8	8	3.842	3.816	0.026	28.971	29.146	−0.175	2.332	2.452	−0.12
8	10	0.8	8	5.362	5.342	0.02	18.726	19.401	−0.675	3.673	3.741	−0.068
9	5	0.6	4	7.163	7.088	0.075	15.936	16.538	−0.602	3.024	3.110	−0.086
10	15	1.0	4	4.913	4.942	−0.029	12.496	12.745	−0.249	1.529	1.630	−0.101
11	5	0.6	8	3.528	3.498	0.03	28.292	28.042	0.25	2.197	2.095	0.102
12	15	1.0	8	6.855	6.930	−0.075	20.235	19.633	0.62	3.789	3.702	0.087
13	10	0.8	6	5.483	5.467	0.016	16.712	18.061	−1.349	3.577	3.597	−0.02
14	10	0.8	6	6.492	5.467	1.025	21.781	18.061	3.72	4.064	3.597	0.467
15	10	0.8	6	4.426	5.467	−1.041	15.691	18.061	−2.73	3.151	3.597	−0.446

**Table 4:** ANOVA of RS, TS and TP after alkali treatment.

RS (mg/mL)	Source	DF	Adj SS	Adj MS	F-Value	*p*-Value
	Regression	9	5.04282	0.560313	21.84	0.002
	Linear	3	1.16342	0.387806	15.11	0.006
	X_1_ (%)	1	0.55469	0.554688	21.62	0.006
	X_2_ (%)	1	0.08226	0.082265	3.21	0.133
	X_3_ (h)	1	0.34699	0.346986	13.52	0.014
	Square	3	1.41322	0.471072	18.36	0.004
	X_1_ × X_1_ (%)	1	0.36356	0.363564	14.17	0.013
	X_2_ × X_2_ (%)	1	0.12917	0.129174	5.03	0.075
	X_3_ × X_3_ (h)	1	0.81202	0.812019	31.64	0.002
	Interaction	3	0.09589	0.031963	1.25	0.386
	X_1_ × X_2_ (%)	1	0.02341	0.023409	0.91	0.383
	X_1_ × X_3_ (h)	1	0.07023	0.070225	2.74	0.159
	X_2_ × X_3_ (h)	1	0.00226	0.002256	0.09	0.779
	Residual Error	5	0.12830	0.025661		
	Lack-of-Fit	3	0.10418	0.034728	2.88	0.268
	Pure Error	2	0.02412	0.012060		
	Total	14	5.17112			
**TS (mg/mL)**	**Source**	**DF**	**Adj SS**	**Adj MS**	**F-Value**	***p*** **-Value**
	Regression	9	274.695	30.5216	5.45	0.038
	Linear	3	97.090	32.3634	5.78	0.044
	X_1_ (%)	1	28.351	28.3515	5.07	0.074
	X_2_ (%)	1	27.498	27.4978	4.91	0.077
	X_3_ (h)	1	22.804	22.8037	4.08	0.100
	Square	3	150.403	50.1343	8.96	0.019
	X_1_ × X_1_ (%)	1	34.278	34.2783	6.13	0.056
	X_2_ × X_2_ (%)	1	20.987	20.9865	3.75	0.111
	X_3_ (h)	1	91.393	91.3930	16.33	0.010
	Interaction	3	21.157	7.0524	1.26	0.382
	X_1_ × X_2_	1	2.304	2.3043	0.41	0.549
	X_1_ × X_3_	1	0.048	0.0477	0.01	0.930
	X_2_ × X_3_	1	18.805	18.8052	3.36	0.126
	Residual Error	5	27.979	5.5958		
	Lack-of-Fit	3	25.729	8.5764	7.63	0.118
	Pure Error	2	2.250	1.1248		
	Total	14	302.67			
**TP (mg/mL)**	**Source**	**DF**	**Adj SS**	**Adj MS**	**F-Value**	***p*** **-Value**
	Regression	9	2.89177	0.321308	8.14	0.016
	Linear	3	0.44948	0.149826	3.80	0.093
	X_1_ (%)	1	0.01257	0.012567	0.32	0.597
	X_2_ (%)	1	0.34304	0.343040	8.69	0.032
	X_3_ (h)	1	0.03269	0.032695	0.83	0.405
	Square	3	0.70544	0.235147	5.96	0.042
	X_1_ × X_1_ (%)	1	0.18887	0.188867	4.78	0.080
	X_2_ × X_2_ (%)	1	0.14052	0.140520	3.56	0.118
	X_3_ × X_3_ (h)	1	0.35008	0.350078	8.87	0.031
	Interaction	3	0.58291	0.194305	4.92	0.059
	X_1_ × X_2_	1	0.03744	0.037442	0.95	0.375
	X_1_ × X_3_	1	0.42576	0.425756	10.79	0.022
	X_2_ × X_3_	1	0.11972	0.119716	3.03	0.142
	Residual Error	5	0.19736	0.039472		
	Lack-of-Fit	3	0.19690	0.065633	284.95	0.003
	Pure Error	2	0.00046	0.000230		
	Total	14	3.08913			

**Table 5:** ANOVA of RS, TS and TP after alkali plus steam treatment.

RS (mg/mL)	Source	DF	Adj SS	Adj MS	F-Value	*p*-Value
	Regression	9	14.6442	1.62714	3.77	0.079
	Linear	3	4.0652	1.35508	3.14	0.125
	X_1_ (%)	1	0.0371	0.03712	0.09	0.781
	X_2_ (%)	1	3.6149	3.61493	8.37	0.034
	X_3_ (h)	1	1.0969	1.09691	2.54	0.172
	Square	3	1.7946	0.59821	1.38	0.349
	X_1_ × X_1_ (%)	1	0.0356	0.03555	0.08	0.786
	X_2_ × X_2_ (%)	1	1.0633	1.06326	2.46	0.178
	X_3_ × X_3_ (h)	1	0.5584	0.55836	1.29	0.307
	Interaction	3	8.1798	2.72659	6.31	0.037
	X_1_ × X_2_ (%)	1	0.2480	0.24800	0.57	0.483
	X_1_ × X_3_ (h)	1	0.1560	0.15602	0.36	0.574
	X_2_ × X_3_ (h)	1	7.7757	7.77573	17.99	0.008
	Residual Error	5	2.1607	0.43213		
	Lack-of-Fit	3	0.0261	0.00870	0.01	0.999
	Pure Error	2	2.1346	1.06728		
	Total	14	16.8049			
**TS (mg/mL)**	**Source**	**DF**	**Adj SS**	**Adj MS**	**F-Value**	***p*-Value**
	Regression	9	324.426	36.0473	7.68	0.019
	Linear	3	23.548	7.8492	1.67	0.287
	X_1_ (%)	1	0.029	0.0290	0.01	0.940
	X_2_ (%)	1	3.778	3.7775	0.80	0.411
	X_3_ (h)	1	15.104	15.1036	3.22	0.133
	Square	3	11.575	3.8582	0.82	0.535
	X_1_ × X_1_ (%)	1	8.252	8.2519	1.76	0.242
	X_2_ × X_2_ (%)	1	4.139	4.1386	0.88	0.391
	X_3_ × X_3_	1	0.053	0.0529	0.01	0.920
	Interaction	3	48.095	16.0315	3.41	0.110
	X_1_ × X_2_	1	0.619	0.6194	0.13	0.731
	X_1_ × X_3_	1	42.146	42.1461	8.98	0.030
	X_2_ × X_3_	1	5.329	5.3292	1.14	0.335
	ResidualError	5	23.473	4.6946		
	Lack-of-Fit	3	2.198	0.7327	0.07	0.971
	Pure Error	2	21.275	10.6376		
	Total	14	347.899			
**TP (mg/mL)**	**Source**	**DF**	**Adj SS**	**Adj MS**	**F-Value**	***p*-Value**
	Regression	9	8.41739	0.93527	9.45	0.012
	Linear	3	0.12669	0.04223	0.43	0.743
	X_1_ (%)	1	0.01412	0.01412	0.14	0.721
	X_2_ (%)	1	0.00378	0.00378	0.04	0.853
	X_3_ (h)	1	0.09963	0.09963	1.01	0.362
	Square	3	1.87128	0.62376	6.30	0.038
	X_1_ × X_1_ (%)	1	0.03397	0.03397	0.34	0.583
	X_2_ × X_2_ (%)	1	0.31951	0.31951	3.23	0.132
	X_3_ × X_3_ (h)	1	1.64965	1.64965	16.67	0.010
	Interaction	3	2.50544	0.83515	8.44	0.021
	X_1_ × X_2_ (%)	1	0.12215	0.12215	1.23	0.317
	X_1_ × X_3_ (h)	1	0.00090	0.00090	0.01	0.928
	X_2_ × X_3_ (h)	1	2.38239	2.38239	24.08	0.004
	Residual Error	5	0.49467	0.09893		
	Lack-of-Fit	3	0.07727	0.02576	0.12	0.938
	Pure Error	2	0.41740	0.20870		
	Total	14	8.91207			

## Data Availability

All the data used for this study is present in this manuscript.
